# Genome-wide association study reveals candidate genes associated with body weight and wool traits in Ordos fine-wool sheep

**DOI:** 10.3389/fvets.2026.1847629

**Published:** 2026-08-28

**Authors:** Lifei Zhang, Ying Gu, Xiaolong He, Biao Wang, Lai Da, Dema De, Rigele Te, Yongbin Liu, Shaoyin Fu

**Affiliations:** 1 Institute of Animal Husbandry, Inner Mongolia Academy of Agricultural & Animal Husbandry Sciences, Hohhot, China; 2 College of Animal Science, Inner Mongolia Agricultural University, Hohhot, China; 3 Ordos Academy of Agricultural & Animal Husbandry Sciences, Ordos City, China

**Keywords:** body weight, candidate genes, GWAS, Ordos fine-wool sheep, sheep breeding, wool fineness, wool length

## Abstract

**Background:**

The Ordos fine-wool sheep is a high-quality fine-wool breed in China, renowned for its excellent wool quality, meat production, and adaptability to the arid and semi-arid regions of Inner Mongolia. Body weight and wool traits are important economic characteristics in sheep breeding. This study aimed to identify genetic loci associated with body weight (BW), wool length (WL), and wool fineness (WF) in Ordos fine-wool sheep.

**Methods:**

A genome-wide association study (GWAS) was conducted in 388 Ordos fine-wool sheep genotyped using the GenoBaits^®^ Ovine 40K SNP panel. Single nucleotide polymorphisms (SNPs) associated with BW, WL, and WF were identified, and candidate genes located near the SNPs reaching the suggestive threshold were subjected to functional annotation and enrichment analysis.

**Results:**

A total of 22 SNPs were identified as potentially associated with BW, WL, and WF traits, corresponding to 27 annotated genes. Functional annotation highlighted six potential candidate genes, including LAMA2, ARHGAP18, IGFBP2, IGFBP5, CA10, and AXIN1, which may play important roles in regulating body weight and wool growth in sheep.

**Conclusions:**

The identified genes provide valuable candidate loci for BW, WL, and WF traits in Ordos fine-wool sheep. The results of this study provide preliminary references for further exploration of the genetic mechanisms of wool traits in Ordos fine-wool sheep and the development of molecular breeding markers.

## 1 Introduction

The Ordos fine-wool sheep is a dual-purpose (wool and meat) breed developed in the Mu Us Desert region of Ordos City, Inner Mongolia Autonomous Region, China. It was bred through long-term selective crossing of local Mongolian sheep with fine-wool breeds such as Xinjiang Merino. This breed is well adapted to the arid and semi-arid environments characterized by sparse vegetation, large temperature fluctuations, and sandy soils. It exhibits strong disease resistance, good grazing ability, and high tolerance to roughage-based diets. The breed is renowned for its high-quality fine wool, tender and

flavorful meat with low fat and cholesterol content, and elastic, soft, thick skin that serves as premium raw material for leather products (1). These traits make the Ordos fine-wool sheep a cornerstone of local animal husbandry and an important economic resource in the region.

To improve these economically important traits through modern molecular breeding, genome-wide association studies (GWAS) have become a powerful tool for identifying marker loci associated with key economic traits across the entire genome. Since its initial development and application to complex diseases (2, 3), GWAS has been widely applied in the fields of animals and plants. GWAS have been established as a powerful tool for the systematic identification and precise localization of quantitative trait loci (QTL) linked to economically significant phenotypes in livestock populations. Numerous GWAS studies have been conducted to locate genetic variants correlated with traits of economic value in animal species (4). In recent years, GWAS have been systematically integrated into modern genetic improvement initiatives for economically vital livestock species, including pigs, cattle, sheep, goats, and chickens (5–9). This approach has facilitated the discovery of functional genomic regions and high-resolution molecular markers associated with economically significant phenotypes (10). Currently, GWAS studies on important traits in sheep have mainly focused on important economic traits such as wool, milk, body weight, meat quality, and reproduction (11–15).

In fine wool sheep production, body weight (BW), wool length (WL), and wool fineness (WF) are critical economic traits that directly determine meat yield, fiber quality, and overall profitability in fine-wool sheep breeding. Body weight serves as a paramount indicator of growth and development, exerting both direct and indirect effects on meat production and wool output, thereby playing a pivotal role in economic returns (16, 17). Wool characteristics, particularly length and fineness, largely determine the quality and commercial value of wool products, making them equally important for the sheep industry. For fine wool sheep breeders, finding genetic markers related to wool characteristics can improve wool quality and thereby enhance the value of the industry. Zhao et al. (4) performed whole-genome sequencing (WGS) on 460 sheep of four fine-wool breeds older than 550 days using the Illumina HiSeq Xten platform. They conducted a GWAS study on eight wool traits of the sequenced sheep using the EMMAX model and ultimately identified 57 SNPs significantly associated with wool characteristics. Within a 100 kb region surrounding these SNPs, a total of 30 candidate genes were identified. With the increasing application of GWAS in fine-wool sheep, numerous genes associated with wool traits have been identified. For example, the *PTPN3* gene is associated with wool yield, while the *SLIT3* and *ZNF280B* genes are associated with wool fiber diameter (18, 19). Additionally, genes such as *KAP6-1, KAP22-1*, and *FST* all influence various wool traits (20–22). Although numerous studies have investigated genes associated with wool traits in fine-wool sheep, these traits are complex quantitative characteristics controlled by multiple genes. However, GWAS studies specifically targeting body weight, wool length, and wool fineness in the Ordos fine-wool sheep, a locally adapted breed with distinct production characteristics and limited genomic resources, have not yet been reported. Comprehensive genetic insights into the polygenic architecture of BW, WL, and WF simultaneously in Ordos fine-wool sheep remain limited. Moreover, major-effect genes and functional markers specifically applicable to molecular breeding in this locally adapted breed are still scarce. Therefore, the present study performed a GWAS using the GenoBaits^®^ Ovine 40K SNP panel in 388 Ordos fine-wool sheep to identify genetic loci and potential candidate genes associated with these three key traits, providing a foundation for MAS and further exploration of the underlying molecular mechanisms in this economically important breed.

## 2 Materials and methods

### 2.1 Experimental animals and phenotypic data collection

**Figure 1:**
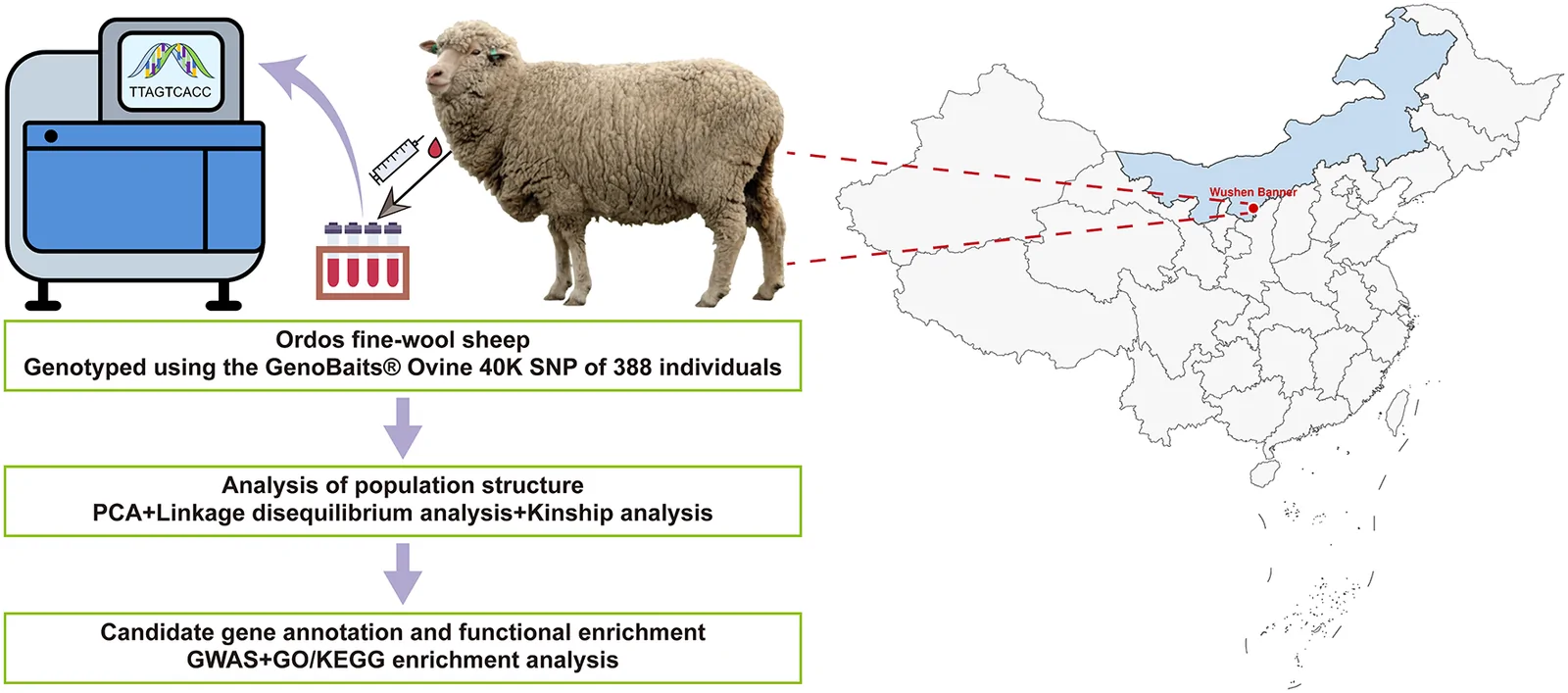
Schematic workflow of the GWAS conducted and geographic location of the sampling sites in Ordos fine-wool sheep. Ordos fine-wool sheep were randomly selected from 10 villages across four townships in Wushen Banner (38.6°N, 108.8°E), Ordos City, Inner Mongolia Autonomous Region, China. A total of 388 individuals were genotyped using the GenoBaits^®^ Ovine 40K SNP panel. Blood samples were collected from the sheep and processed for genotyping. After quality control and genotyping, population structure was analyzed using PCA, LD decay analysis, and kinship analysis. Subsequently, GWAS was performed using the FarmCPU model, followed by candidate gene annotation within ±50 kb of SNPs reaching the suggestive threshold and functional enrichment analysis (GO and KEGG).

A total of 388 Ordos fine-wool sheep were included in this study, comprising 30 males and 358 females. The sheep were randomly selected from 10 villages across four townships in Wushen Banner, Ordos City, Inner Mongolia Autonomous Region, China. No pedigree information was available for the selected animals. All individuals were adults, ranging in age from 1 to 6 years old. Phenotypic measurements were conducted during the regular spring shearing season (early to mid-May) by trained technicians following a standardized protocol. Three phenotypic traits were recorded for all 388 sheep: body weight (BW), wool length (WL), and wool fineness (WF). BW was measured using an electronic scale after overnight fasting. WL was measured at the mid-side shoulder site on unshorn fleece. WF was graded in accordance with the Chinese national standard GB 1523-2013 (Wool of Sheep), which classifies wool fineness using the Bradford spinning count(s) system. Under this standard, a grade of ≥66s corresponds to a mean fiber diameter of approximately ≤ 20.0 μm and is designated as high-quality fine wool, the threshold routinely applied in Chinese fine-wool sheep breeding programs and commercial wool procurement. Although the sheep originated from different villages, they were raised under a uniform semi-pastoral management system with consistent supplemental feeding during winter, thereby minimizing environmental variability. Age was included as a covariate in subsequent analyses to account for physiological differences. Blood samples (5 ml) were collected from the jugular veins of all sheep and stored in EDTA anticoagulant tubes at −20 °C for subsequent genotyping using a GenoBaits^®^ Ovine 40K liquid-phase SNP chip. Genomic DNA was extracted from blood samples using the TIANamp Blood DNA Kit (TIANGEN Biotech Co., Ltd., Beijing, China) following the manufacturer's protocol. DNA concentration was quantified using a NanoDrop 2,000 spectrophotometer (Thermo Fisher Scientific, USA), and DNA integrity was assessed by 1% agarose gel electrophoresis. Only samples with OD260/280 ratios between 1.8 and 2.0 and a concentration ≥50 ng/μL were submitted for genotyping. Descriptive statistical analysis of the phenotype and analysis pipeline is presented in Figure 1.

WF is a key economic trait in fine-wool sheep and is routinely classified in accordance with Chinese industry standards. In this study, WF was treated as a binary trait: fibers with a grade of ≥66s (high-quality fine wool) were coded as one, and those < 66s as zero. This classification aligns with the practical grading system applied in commercial wool production and breeding programs. Although dichotomizing a potentially continuous trait (fiber diameter) entails some loss of information, this approach is commonly adopted in livestock GWAS when only categorical industry grades are available. Descriptive statistics and chi-square analyses for this binary trait were performed, including the calculation of proportions with corresponding 95% confidence intervals (CI), using the Wilson score method via the binom package. A chi-square goodness-of-fit test was conducted to assess the deviation from an equal distribution. All statistical analyses for the binary wool fineness trait were performed in R (version 4.4.3), with data visualization generated using the ggplot2 package.

### 2.2 Genotyping and genotype data quality control

Genotyping was performed using the GenoBaits^®^ Ovine 40K Liquid-Phase SNP Chip (MolBreeding Biotech Ltd., Shijiazhuang, China). The Oar_v4.0 assembly of the sheep reference genome, sourced from the NCBI database, was used for alignment. Oar_v4.0 was selected because the probe sequences of the GenoBaits^®^ Ovine 40K SNP chip are designed and annotated based on this assembly, ensuring coordinate consistency throughout the analysis pipeline. Genotyping data underwent quality control (QC) processing using Plink (version 1.90), which involved a total of 40,736 SNPs (23). SNPs with a missingness rate >2%, Hardy-Weinberg equilibrium (HWE) *p*-values < 1 × 10^−6^, or minor allele frequency (MAF) < 5% were excluded from the analysis. Individual samples with >10% missing genotypes were also excluded. These thresholds are consistent with standard practice in livestock GWAS: the MAF ≥5% cutoff reduces noise from rare variants and improves statistical power; the HWE filter removes loci potentially affected by genotyping errors or population stratification; and the missingness thresholds ensure data completeness for reliable association analysis. After stringent quality filtering of both SNPs and individuals, the high-quality data were used for downstream GWAS. The identity by state (IBS) matrix and the genomic relatedness matrix (G matrix) were constructed using Plink (version 1.90), and kinship results were visualized in R (version 4.4.3). In the absence of pedigree data, the IBS matrix was used as a genomic surrogate for identity-by-descent (IBD) analysis to assess cryptic relatedness among sampled individuals. Principal component analysis (PCA) was performed with the rMVP package, using the filePC = TRUE parameter to output principal components for adjusting population stratification (24). The resulting principal components were saved in the MVP. pc.desc file, and the PCA results were visualized in R using the ggplot2 package and outputs from rMVP. Linkage disequilibrium (LD) decay analysis was conducted with PopLDdecay (version 3.40) to calculate the LD coefficient (r^2^) for paired SNPs (25). The raw genotyping datasets of 388 Ordos fine-wool sheep have been deposited in the Genome Variation Map (GVM) (26) in National Genomics Data Center, Beijing Institute of Genomics, Chinese Academy of Sciences and China National Center for Bioinformation (27), under accession number GVM001404.

### 2.3 Genome-wide association studies model

Using rMVP software (version 1.4.0, https://github.com/xiaolei-lab/rMVP), we conducted GWAS for body weight, wool length, and wool fineness using the FarmCPU model (24). Although FarmCPU was originally developed for quantitative traits, linear mixed models have been widely and successfully applied to binary traits in livestock GWAS. The FarmCPU model addresses a critical challenge in GWAS: enhancing statistical power to detect causal variants while controlling for false positives due to population structure. It incorporates a fixed-effects model to effectively control confounding effects from population structure and kinship, thus improving the accuracy and robustness of the association analysis. Unlike traditional methods, FarmCPU eliminates the need to collect kinship information for all markers or related labels. Instead, it constructs a kinship matrix based on markers significantly associated with the traits and uses a maximum likelihood estimation strategy to screen and optimize related markers. This approach overcomes limitations of the stepwise regression model. Through iterative computation, FarmCPU combines the benefits of fixed-effect and random-effect models, effectively controlling for confounding factors and efficiently detecting genetic variation. In summary, FarmCPU offers both statistical power and computational efficiency for GWAS. The model architecture is represented as follows:


$$\begin{array}{lll} y_{i} & = & M_{i1}b_{1}+M_{i2}b_{2}+...+M_{in}b_{n}+Z_{ij}u_{j}+e_{i} \end{array}$$ (1)

$$\begin{array}{lll} y_{i} & = & V_{i}+e_{i} \end{array}$$ (2)

In Equations 1 and 2, Equation 1 is a fixed-effects model that includes sex, age, villages, and the first three principal components (nPC.FarmCPU = 3) as covariates to correct for population stratification, while Equation 2 is a random-effects model. *y*_*i*_ indicates the phenotypic observation of the *i*-th individual; *M*_*i*1_ signifies the genotypes of n potential correlation loci included in the model; *b*_*n*_ represents the effect value of the loci with potential associations incorporated into the model; *Z*_*ij*_ denotes the genotype of the *j*−th marker of the *i*−th individual; *u*_*j*_ indicates the effect value of the genotype at the corresponding locus of *Z*_*ij*_; *V*_*i*_ represents the total genetic effect of the *i*−th individual, and *e*_*i*_ is the residual vector, subject to $e\sim N\left(0,\;I\sigma_{e}^{2}\right)$. When executing the FarmCPU model, styles (Equations 1 and 2) are alternate operations.

To minimize false-positive associations, a suggestive significance threshold (1/N_snp_) was applied to the GWAS findings, as the conventional Bonferroni correction would be overly stringent for this dataset. Here, N_snp_ represents the post-QC count of polymorphic markers (28).

### 2.4 Bioinformatic analysis

Genetic information from the 50 kb upstream and downstream flanking regions of each SNP reaching the suggestive threshold was retrieved using Bedtools software (version 2.31.1) (29). Genes associated with SNPs reaching the suggestive threshold were annotated using the sheep reference genome (Oar_v4.0, GCF_000298735.2) and analyzed for gene ontology (GO) and Kyoto encyclopedia of genes and genomes (KEGG) enrichment using the R package clusterProfiler (30). Functional enrichment analysis was performed with the enrichGO and enrichKEGG functions. Terms with a *p*-value < 0.05 were considered statistically significant and were retained for visualization.

## 3 Results

### 3.1 Phenotypic data analysis of body weight and wool traits

**Figure 2:**
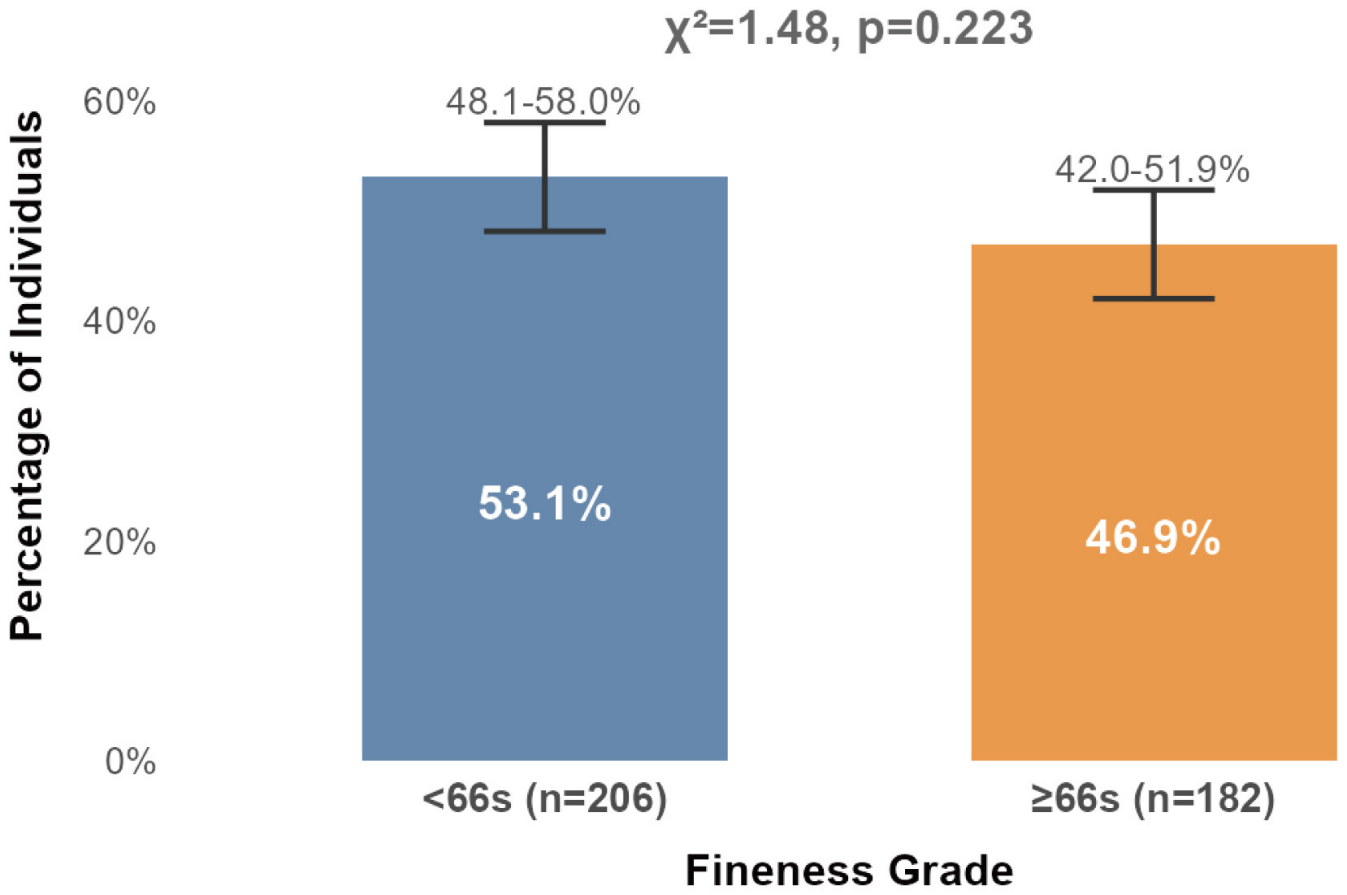
Distribution of wool fineness grades in Ordos fine-wool sheep, showing the percentage of individuals meeting the ≥66s standard and those below this threshold. Error bars represent 95% confidence intervals.

We performed descriptive statistical analysis on the phenotypic data of 388 Ordos fine-wool sheep. Table 1 presents the descriptive statistics for BW and WL, including the mean, maximum, minimum, standard error, and coefficient of variation (CV). The mean body weight (BW) was 51.30 ± 16.36 kg, and the mean wool length (WL) was 10.36 ± 1.46 cm. For wool fineness (WF), 46.9% (95% CI: 42.0–51.9%) of the sheep produced wool meeting the ≥66s industry standard. These values are consistent with the typical phenotypic characteristics of Ordos fine-wool sheep, reflecting favorable growth performance and wool quality adapted to the local environment. The CV ranged from 14.08% to 31.88% across the measured traits. The 30 rams in the study, all of which were breeding males, had significantly higher body weights than the ewes, contributing to an elevated maximum value, increased standard deviation, and higher CV for body weight in the overall sample. In addition, the sampled animals spanned a wide age range (1–6 years), which also contributed to the elevated CV for body weight, as live weight increases substantially with physiological maturation in sheep. Both sex and age were included as fixed effects in the GWAS model to account for these non-genetic sources of variation. Analysis of the binary wool fineness trait revealed that 46.9% (95% CI: 42.0–51.9%) of the sheep produced wool meeting the ≥66s standard. Chi-square analysis showed no significant deviation from an equal distribution (χ^2^ = 1.48, df = 1, *p* = 0.223), indicating balanced wool fineness characteristics in this population. The statistical analysis for wool fineness is presented in Figure 2.

**Table 1:** Descriptive statistics of body weight and wool length traits.

Item	Number	Mean	Max	Min	SD	CV (%)
BW (kg)	388	51.30	113	27.2	16.36	31.88
WL (cm)	388	10.36	16	7	1.46	14.08

BW, body weight; WL, wool length; SD, standard deviation; CV, coefficient of variation.

### 3.2 Data quality control and population genetic analysis

**Figure 3:**
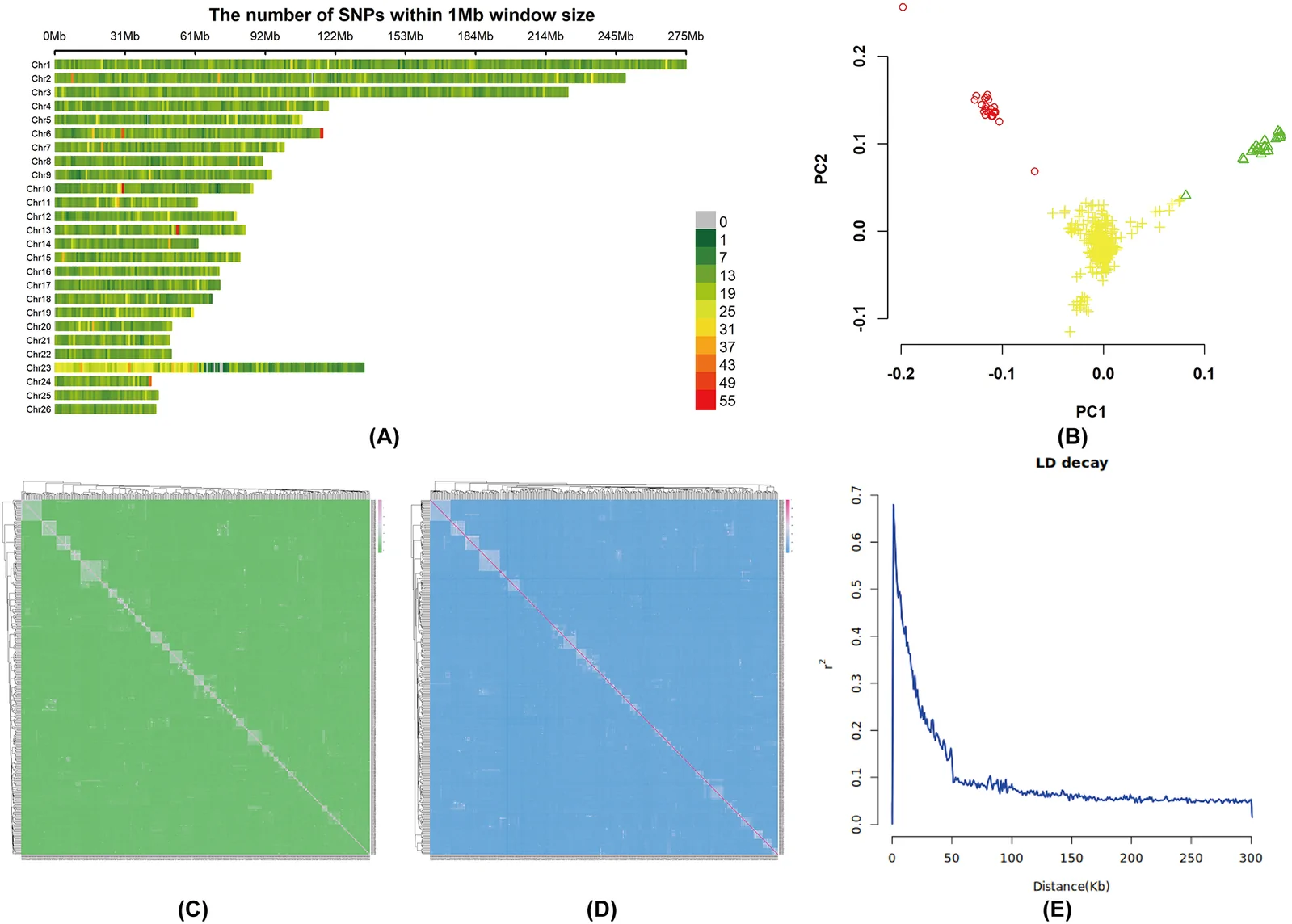
Quality control and population genetic structure of the Ordos fine-wool sheep population. **(A)** Distribution of the number of SNPs within 1 Mb windows across the 26 autosomes. **(B)** PCA of the population. **(C)** Heatmap of the genomic relationship matrix (G matrix). **(D)** Heatmap of the identity-by-state (IBS) distance matrix. **(E)** LD decay plot across the genome.

QC initially included 40,736 SNPs. After applying stringent filtering criteria (geno < 0.02, MAF > 0.05, HWE *p* > 1 × 10^−6^, mind > 0.1), a total of 35,819 high-quality SNPs were retained and mapped across all 26 sheep autosomes (Figure 3A). These SNPs were subsequently used for PCA. The PCA results (Figure 3B) revealed population stratification, and therefore, the principal components were included as covariates in the GWAS model. To further assess genetic relationships among individuals, a genomic relationship matrix (G matrix) was constructed using genome-wide markers. The heatmap of the G matrix (Figure 3C) showed that the kinship among individuals was generally moderate to low, suggesting a low level of inbreeding within the Ordos fine-wool sheep population. The corresponding IBS matrix (Figure 3D) further illustrated that most individuals exhibited moderate to low genetic relatedness, with only a few pairs showing higher similarity, which was consistent with the G matrix results. Linkage disequilibrium (LD) decay analysis showed that LD decayed rapidly and the r^2^ value dropped to approximately 0.1 at a genomic distance of 50 kb in Ordos fine-wool sheep (Figure 3E). Based on this result, genes located within ±50 kb of SNPs reaching the suggestive threshold were considered potential candidate genes. Previous studies have also reported relatively low LD levels in sheep populations, with an r^2^ value of 0.1 typically corresponding to a genetic distance of approximately 50 kb (31).

### 3.3 Genome-wide association study

**Figure 4:**
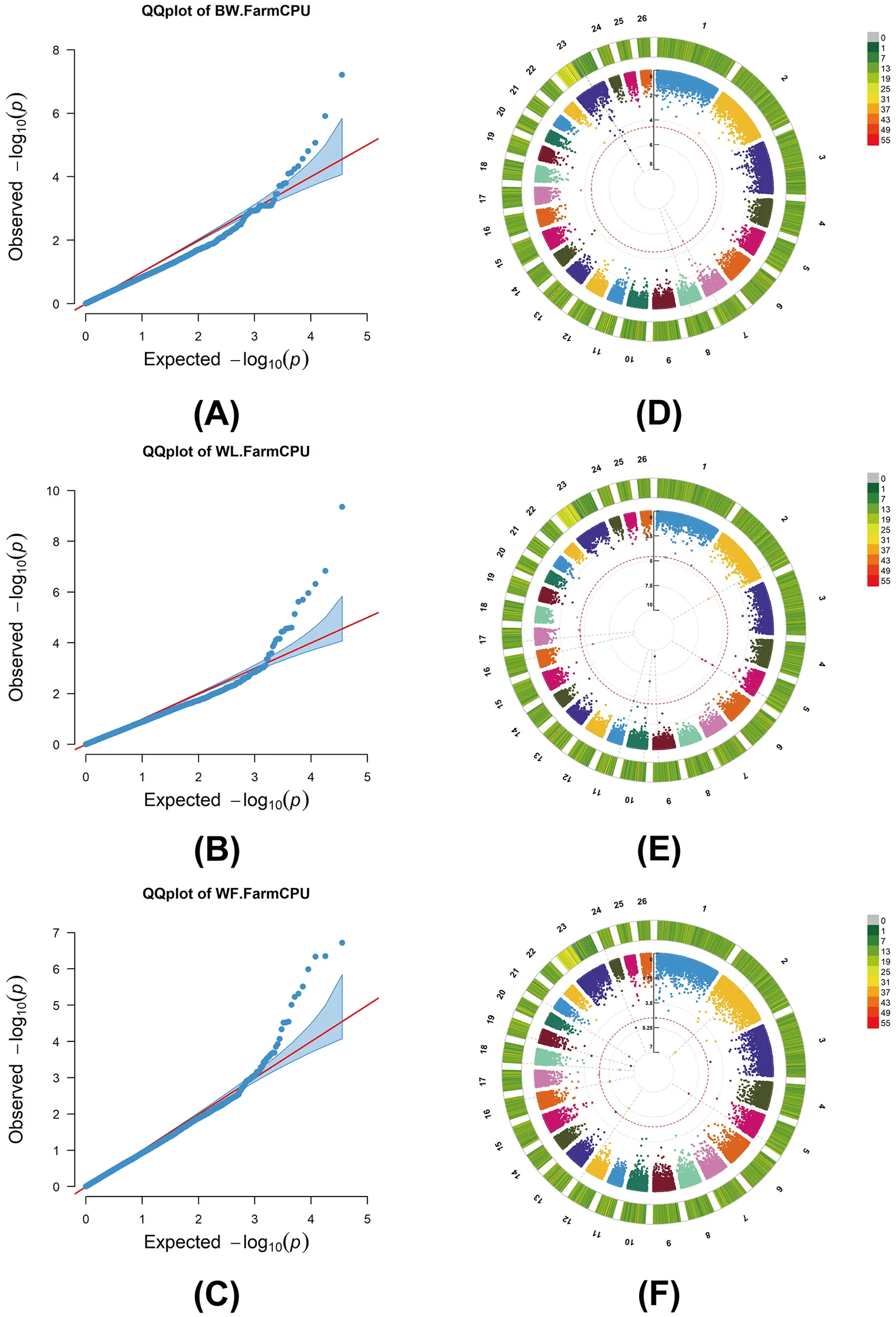
QQ plots and Manhattan plots showing GWAS results for BW, WL, and WF in Ordos fine-wool sheep. **(A–C)** QQ plots for BW, WL, and WF, respectively. **(D–F)** Corresponding Manhattan plots. The red dotted line indicates the suggestive significance threshold (1/N_snp_). SNPs exceeding this threshold are considered potentially associated loci.

To reduce false-positive associations, sex, age, and villages were included as fixed effects in the model. In addition, due to the presence of group stratification, this study included the principal components from the PCA as covariates in the model. The FarmCPU model was used to perform GWAS for BW, WL, and WF. The Manhattan plots and quantile–quantile (QQ) plots are presented in Figure 4. Although the conventional Bonferroni correction is statistically more stringent, a suggestive significance threshold of 1/N_snp_ was applied in this study, which is a common practice in livestock GWAS with moderate sample sizes. Using the Bedtools software (version 2.31.1), we annotated genomic regions within a ±50 kb range around SNPs that met the suggestive significance threshold to 27 genes (Table 2). For BW, five SNPs reaching the suggestive threshold were detected on Chr7, Chr8, and Chr23, corresponding to three candidate genes (Figure 4D; Table 2). For WL, nine SNPs reaching the suggestive threshold were identified on Chr2, Chr5, Chr9, Chr10, Chr11, Chr16, and Chr17, corresponding to seven candidate genes (Figure 4E; Table 2). For WF, eight SNPs reaching the suggestive threshold were detected on Chr2, Chr5, Chr12, Chr16, Chr18, Chr19, Chr21, and Chr24, corresponding to 17 candidate genes (Figure 4F; Table 2). As shown in Figure 4, the observed *p*-values deviated from the expected distribution at lower *p*-values (below 10^−4^), suggesting the presence of true genetic associations. Through functional annotation and literature review, six potential candidate genes related to growth and wool traits in Ordos fine-wool sheep were identified. These included (laminin subunit alpha-2) *LAMA2* and (Rho GTPase-activating protein 18) *ARHGAP18* associated with BW; (insulin-like growth factor binding protein 2) *IGFBP2*, (insulin-like growth factor binding protein 5) *IGFBP5*, and (carbonic anhydrase 10) *CA10* associated with WL; and (axis inhibition protein 1) *AXIN1* associated with WF.

**Table 2:** Description of SNPs reaching the suggestive significance threshold and potential candidate genes of body weight and wool traits in Ordos fine-wool sheep.

Trait	Chr	SNP name	Position	Ref	Alt	*P*-value	Gene name	Gene position
BW	7	chr7_74129297	74129297	T	C	1.56E-05	*MAX*	74060187-74083840
	8	chr8_54843055	54843055	A	G	2.74E-05	*ARHGAP18*	54888688-55088508
	8	chr8_54843055	54843055	A	G	2.74E-05	*LAMA2*	54315640-54842723
	23	chr23_78674874	78674874	G	T	1.22E-06	–	–
	23	chr23_79845760	79845760	T	C	6.18E-08	–	–
	23	chr23_82464920	82464920	C	T	8.56E-06	–	–
WL	2	chr2_217608419	217608419	T	C	2.42E-06	*IGFBP2*	217602922-217632202
	2	chr2_217608419	217608419	T	C	2.42E-06	*IGFBP5*	217641543-217664673
	5	chr5_83976570	83976570	T	C	7.34E-06	–	–
	5	chr5_86600075	86600075	C	T	1.10E-06	–	–
	5	chr5_87229402	87229402	A	G	4.75E-07	*LOC105615382*	87175629-87180686
	5	chr5_87229402	87229402	A	G	4.75E-07	*CETN3*	87209050-87228546
	9	chr9_72871293	72871293	C	A	4.41E-10	*RIMS2*	72847843-73449938
	10	chr10_12027818	12027818	A	G	1.48E-07	*VWA8*	12023184-12413423
	11	chr11_579065	579065	C	G	2.56E-05	*CA10*	268450-1109337
	16	chr16_47059495	47059495	T	A	2.01E-06	–	–
	17	chr17_22088874	22088874	T	C	2.64E-05	–	–
WF	2	chr2_100659711	100659711	G	C	4.47E-07	*TMEM215*	100696899-100698894
	5	chr5_92361883	92361883	C	T	3.07E-06	*GLRX*	92397349-92397464
	5	chr5_92361883	92361883	C	T	3.07E-06	*RHOBTB3*	92306671-92368456
	12	chr12_78751926	78751926	G	A	4.82E-06	*NAV1*	78695539-78815105
	16	chr16_56663242	56663242	C	A	5.98E-06	–	–
	18	chr18_2372749	2372749	T	C	1.02E-06	–	–
	19	chr19_11778693	11778693	T	G	1.93E-07	*XYLB*	11725660-11766807
	19	chr19_11778693	11778693	T	G	1.93E-07	*EXOG*	11815457-11835432
	19	chr19_11778693	11778693	T	G	1.93E-07	*ACVR2B*	11794561-11812287
	21	chr21_161925	161925	A	C	4.64E-07	*HEPHL1*	156077-247143
	21	chr21_161925	161925	A	C	4.64E-07	*PANX1*	94263-149584
	24	chr24_269627	269627	C	T	9.76E-06	*RGS11*	220100-226561
	24	chr24_269627	269627	C	T	9.76E-06	*ARHGDIG*	230679-232285
	24	chr24_269627	269627	C	T	9.76E-06	*PDIA2*	232487-235507
	24	chr24_269627	269627	C	T	9.76E-06	*MRPL28*	286815-289463
	24	chr24_269627	269627	C	T	9.76E-06	*NME4*	312296-315551
	24	chr24_269627	269627	C	T	9.76E-06	*DECR2*	316930-323684
	24	chr24_269627	269627	C	T	9.76E-06	*TMEM8A*	290815-295902
	24	chr24_269627	269627	C	T	9.76E-06	*AXIN1*	235832-272664

BW, body weight; WL, wool length; WF, wool fineness.

### 3.4 Functional enrichment analysis of candidate genes

**Figure 5:**
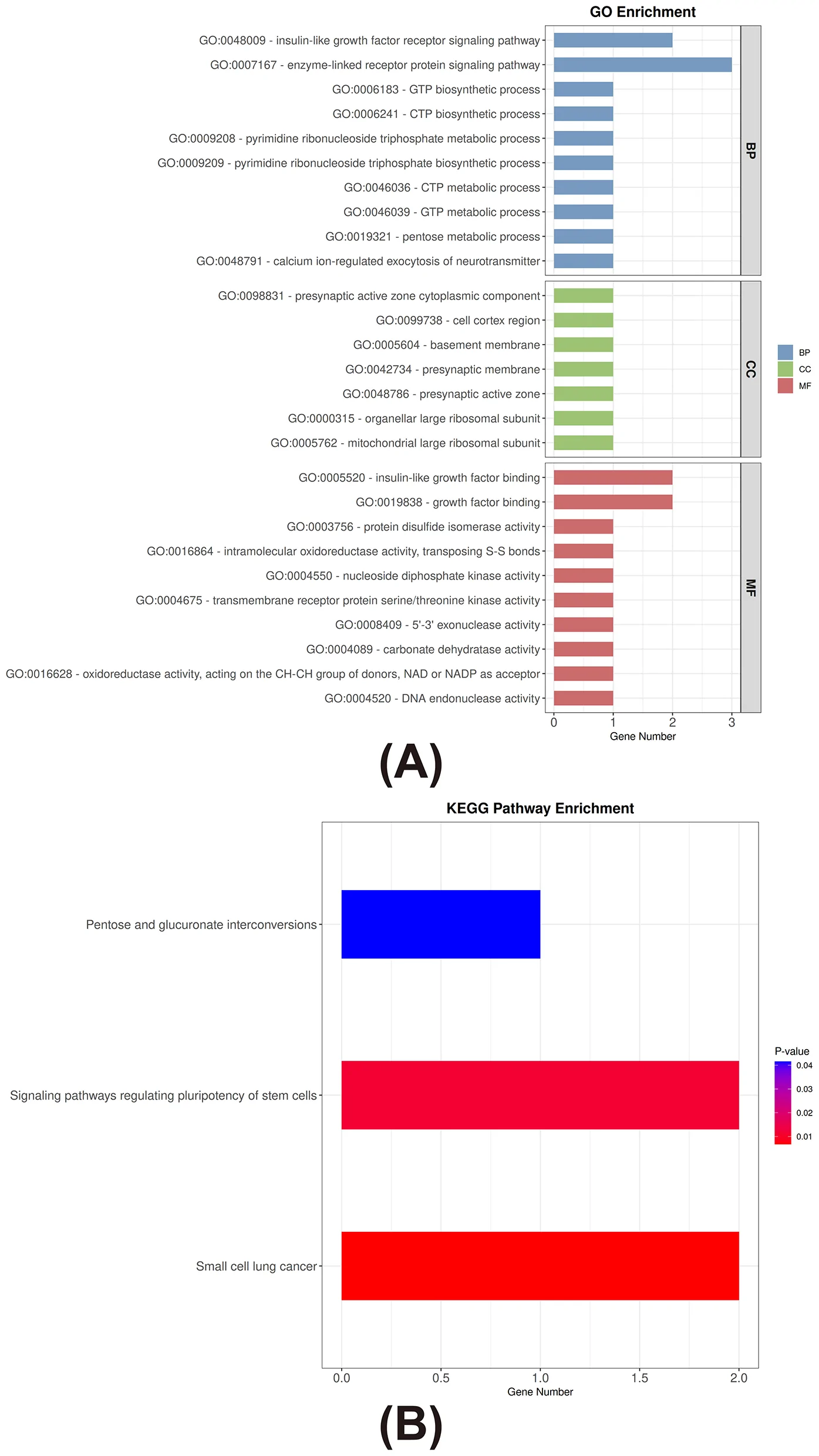
Functional enrichment analysis of the 27 candidate genes. **(A)** GO enrichment analysis showing biological process (BP), cellular component (CC), and molecular function (MF) categories. **(B)** KEGG pathway enrichment analysis.

To further explore the biological functions of the genes associated with SNPs reaching the suggestive threshold, functional enrichment analyses were performed on the annotated candidate genes. GO analysis suggested that these genes might be associated with several biological processes (Figure 5A), enzyme-linked receptor protein signaling pathway (GO:0007167), insulin-like growth factor receptor (GO:0048009), GTP biosynthetic process (GO:0006183), and CTP biosynthetic process (GO:0006241). In terms of cellular components, the candidate genes appeared to be enriched in presynaptic active zone (GO:0048786), basement membrane (GO:0005604), and cell cortex region (GO:0099738). At the molecular function level, enrichment was observed in insulin-like growth factor binding (GO:0005520) and growth factor binding (GO:0019838). KEGG pathway analysis revealed that these genes were primarily enriched in pathways related to stem cell regulation and disease (Figure 5B). The most significantly enriched pathways included signaling pathways regulating pluripotency of stem cells and small cell lung cancer. It should be noted that the appearance of disease-related pathways such as small cell lung cancer reflects shared molecular components (e.g., *LAMA2* and *AXIN1*) annotated across multiple KEGG pathways derived from human disease databases, rather than indicating direct disease relevance in sheep. Additionally, a metabolic pathway, pentose and glucuronate interconversions, was also identified. These results suggest that the candidate genes may play roles in cell differentiation, proliferation, and basic metabolic processes.

## 4 Discussion

The Ordos fine-wool sheep is a dual-purpose breed developed in China that is well adapted to the cold and semi-arid environments of the Inner Mongolia Plateau, where it survives on sparse vegetation and tolerates large temperature fluctuations. This breed is characterized by high-quality wool and good meat production performance. Most wool traits in fine-wool sheep are quantitative traits regulated by complex genetic mechanisms (32). Body weight is an important economic trait in sheep production; therefore, understanding its underlying molecular mechanisms and identifying key functional genes are essential for improving sheep breeding programs. Wool fineness and wool length are also important indicators of wool quality and economic value, with fineness being the most critical factor. In general, finer wool combined with greater fiber length and yield contributes to higher wool quality and economic value. Consequently, extensive studies have focused on improving wool production and fiber quality. Identifying candidate genes associated with wool traits is therefore essential for fine-wool sheep breeding. In this study, GWAS was conducted using genotype data generated from the GenoBaits^®^ Ovine 40K liquid-phase SNP chip on 388 Ordos fine-wool sheep from Wushen Banner, Ordos City. The analysis aimed to identify candidate genes associated with body weight, wool length, and wool fineness. Although the study population had a highly unbalanced sex ratio and animals were sampled from 10 villages across 4 townships, several approaches were employed to control for potential confounding. Sex, age, and village were fitted as fixed effects, and PCA was used to correct for population stratification (33). The results of this study indicate that the loci identified in the GWAS analysis are consistent whether villages are included as covariates or excluded; this demonstrates that the genetic signals identified in this study are relatively reliable. After model correction, the QQ plots indicated a good fit between the observed and expected distributions. It should be noted that the choice of multiple-testing correction in GWAS is often context-dependent. While the classical Bonferroni correction is statistically conservative, it can be overly stringent for studies with moderate sample sizes. The suggestive significance threshold of 1/N_snp_ adopted in the present study is a widely accepted standard in sheep GWAS research.

This study identified *LAMA2* and *ARHGAP18* as potential candidate genes associated with body weight in Ordos fine-wool sheep. Previous studies have reported that the *LAMA2* gene is involved in pig oocyte development (34). Our findings further suggest that this gene may also be involved in regulating growth traits in sheep. *LAMA2* is an essential component of the extracellular matrix (ECM) and was significantly enriched in GO terms related to the basement membrane (GO:0005604) and extracellular matrix structural constituent (GO:0005201), which are critical for maintaining ECM integrity and cellular signaling (35). As a core component of the ECM–receptor interaction pathway, *LAMA2* likely plays a vital role in maintaining skeletal muscle stability and transmitting mechanical signals (36, 37). Mutations in *LAMA2* have also been reported to cause congenital muscular dystrophy (38). For *ARHGAP18*, our results showed its involvement in the small GTPase-mediated signal transduction pathway (GO:0007264). *ARHGAP18* belongs to the Rho GTPase-activating protein family and plays an important role in regulating intracellular signal transduction. It acts as a GTPase-activating protein for RhoA, thereby regulating cell morphology, migration, and cytoskeletal dynamics (39). In addition, *ARHGAP18* has been reported to function as a regulator of vascular formation (40). Hatzirodos et al. suggested that *ARHGAP18* may participate in the growth of bovine follicular granulosa cells (41). Similar studies in Boer goats have shown that members of the *ARHGAP* family genes are involved in cytoskeletal regulation, cell proliferation, and differentiation in muscle tissue (42). Consistent with these findings, our results suggest that *ARHGAP18* may be a potential candidate gene influencing body weight traits in Ordos fine-wool sheep. Therefore, we speculate that *LAMA2* and *ARHGAP18* may influence body weight by regulating ECM organization and skeletal muscle development.

For the wool length trait, *IGFBP2* and *IGFBP5* are known to play central roles in the insulin-like growth factor (IGF) signaling pathway, which regulates cell proliferation and differentiation (43). In this study, these genes were significantly enriched in GO terms, including the insulin-like growth factor receptor signaling pathway (GO:0048009) and enzyme-linked receptor protein signaling pathway (GO:0007167). These findings are consistent with their established roles as IGF-binding proteins that regulate IGF bioavailability and downstream signaling. The IGF signaling pathway is known to play a key role in hair follicle development, influencing keratinocyte proliferation, follicle morphogenesis, epidermal regeneration, and the anagen phase of the hair cycle (44–46). Therefore, *IGFBP2* and *IGFBP5* may influence wool follicle development and contribute to variation in wool length. The *CA10* gene, which encodes carbonic anhydrase X, was enriched in carbonate dehydratase activity (GO:0004089). Carbonic anhydrases play important roles in maintaining pH homeostasis in biological tissues. This activity may regulate the microenvironment of wool follicles by maintaining optimal conditions for enzymatic activity and cell proliferation. Previous studies have demonstrated that changes in the skin microenvironment can influence hair follicle development and mammalian hair growth (47). Although direct evidence linking *CA10* to wool follicle biology is currently limited, its potential role in regulating local pH suggests that it may indirectly influence wool growth through modulation of the follicular microenvironment.

For the wool fineness trait, *AXIN1* emerged as a potential candidate gene. The Wnt/β-catenin signaling pathway plays a critical role in hair follicle morphogenesis and regeneration (48). For instance, miR-181a-5p in Hu sheep exosomes has been shown to activate this pathway by targeting WIF1, thereby promoting follicle growth (49), with similar mechanisms reported in rabbits (50). Given that *AXIN1* is a core negative regulator of the Wnt pathway (51), it may participate in regulating hair follicle morphogenesis and epithelial–mesenchymal interactions, potentially by inhibiting Wnt signaling as suggested by previous studies (52). These findings provide important insights for future studies on major genes affecting wool traits and for the development of genetic markers in sheep breeding. A limitation of the present study is the use of a binary phenotype for wool fineness. While this approach aligns with industry grading standards and facilitates direct application in breeding programs, it discards information that continuous fiber diameter measurements could provide and may reduce statistical power. Future GWAS using high-precision fiber diameter data would complement the current findings. Nevertheless, the identification of *AXIN1*, a key regulator of the Wnt/β-catenin pathway that influences hair follicle development, supports the biological relevance of the detected signals. It should be noted that the candidate genes reported in this study were annotated based on their proximity (±50 kb) to SNPs reaching the suggestive threshold, guided by the LD decay pattern observed in this population. While this is a standard approach in GWAS with medium-density SNP panels, proximity does not prove causality. The six genes highlighted were prioritized based on their functional annotations, involvement in relevant biological pathways, and supporting evidence from previous studies in sheep or other mammals. Thus, these represent plausible candidates that warrant further validation through fine-mapping, expression profiling, or functional assays. Additionally, the highly unbalanced sex ratio in this study represents a structural limitation. Although sex was fitted as a fixed effect in the model, the small male subgroup (*n* = 30) precluded sex-stratified analyses, and residual confounding from sexual dimorphism or sex-linked loci cannot be fully excluded. Future studies should aim to include a more balanced sex representation to better disentangle sex-specific genetic effects.

## 5 Conclusion

This study conducted a GWAS in 388 Ordos fine-wool sheep using a 40K SNP panel and the FarmCPU model to investigate the genetic basis of BW, WL, and WF. We identified 22 SNPs reaching the suggestive significance threshold, annotating 27 positional candidate genes within ±50 kb flanking regions. Among these, six genes (*LAMA2, ARHGAP18, IGFBP2, IGFBP5, CA10*, and *AXIN1*) were identified as plausible candidate genes that we speculate may be associated with growth and wool traits. Exploratory functional enrichment analysis suggested possible involvement of these genes in biological processes related to the ECM organization and growth factor signaling. These results provide valuable preliminary insights and generate useful hypotheses for the molecular mechanisms underlying important economic traits in Ordos fine-wool sheep. While further validation in larger cohorts is warranted, these findings establish a foundation for MAS to enhance growth and wool quality in Ordos fine-wool sheep. Specifically, the identified SNPs in *LAMA2* and *IGFBP* genes represent promising targets for early genetic screening. Future efforts should focus on validating these markers in independent commercial flocks and employing functional assays including tissue-specific expression profiling, eQTL analysis, and *in vitro*/*in vivo* perturbation of the candidate genes to elucidate the underlying regulatory mechanisms.

## Data Availability

The datasets presented in this study can be found in online repositories. The genome variation data have been deposited in the Genome Variation Map (GVM) in the National Genomics Data Center, China National Center for Bioinformation, under accession number GVM001404, associated with BioProject PRJCA063378, https://ngdc.cncb.ac.cn/gvm/getProjectDetail?Project=GVM001404.

## Supplementary material

**Supplementary Table S1**

GO analysis for the regional candidate genes associated with SNPs reaching the suggestive significance threshold.

**Supplementary Table S2**

KEGG analysis for the regional candidate genes associated with SNPs reaching the suggestive significance threshold.
